# Dynamic changes in DNA demethylation in the tree shrew (*Tupaia belangeri chinensis*) brain during postnatal development and aging

**DOI:** 10.24272/j.issn.2095-8137.2017.013

**Published:** 2017-03-18

**Authors:** Shu Wei, Hai-Rong Hua, Qian-Quan Chen, Ying Zhang, Fei Chen, Shu-Qing Li, Fan Li, Jia-Li Li

**Affiliations:** ^1^Key Laboratory of Animal Models and Human Disease Mechanisms of the Chinese Academy of Sciences & Yunnan Province, Kunming Institute of Zoology, Chinese Academy of Sciences, Kunming Yunnan 650223, China; ^2^Kunming College of Life Science, University of Chinese Academy of Sciences, Kunming Yunnan 650223, China; ^3^Department of Pathology and Pathophysiology, School of Basic Medical Science, Kunming Medical University, Kunming Yunnan 650500, China; ^4^School of Life Science, University of Science and Technology of China, Hefei Anhui 230027, China; ^5^Kunming Primate Research Center of the Chinese Academy of Sciences, Kunming Institute of Zoology, Chinese Academy of Sciences, Kunming Yunnan 650223, China

**Keywords:** Tree shrew, DNA demethylation, 5-hydroxymethylcytosine, Brain development and aging

## Abstract

Brain development and aging are associated with alterations in multiple epigenetic systems, including DNA methylation and demethylation patterns. Here, we observed that the levels of the 5-hydroxymethylcytosine (5hmC) ten-eleven translocation (TET) enzyme-mediated active DNA demethylation products were dynamically changed and involved in postnatal brain development and aging in tree shrews (*Tupaia belangeri chinensis*). The levels of 5hmC in multiple anatomic structures showed a gradual increase throughout postnatal development, whereas a significant decrease in 5hmC was found in several brain regions in aged tree shrews, including in the prefrontal cortex and hippocampus, but not the cerebellum. Active changes in Tet mRNA levels indicated that TET2 and TET3 predominantly contributed to the changes in 5hmC levels. Our findings provide new insight into the dynamic changes in 5hmC levels in tree shrew brains during postnatal development and aging processes.

## INTRODUCTION

Epigenetic systems emphasize the heritable changes in gene expression that do not involve coding sequence modifications, e.g., DNA methylation, histone modification and chromatin remodeling, and non-coding RNA regulation. These types of changes are proposed to be responsible for controlling the expression and function of genes and have emerged as important mediators of development and aging (Abel & Zukin, 2008; Agis-Balboa et al., 2013; Alagiakrishnan et al., 2012; Bakulski et al., 2012; Barbash & Soreq, 2012; Bihaqi et al., 2012; Coppieters & Dragunow, 2011; Deaton & Bird, 2011). Complex disorders, such as cardiovascular disease, cancer, diabetes, and neuropsychiatric and neurodegenerative diseases (Kinney & Pradhan, 2013; Konsoula & Barile, 2012; Kudo et al., 2012; Kwok, 2010; Maekawa & Watanabe, 2007; Marques et al., 2011; Urdinguio et al., 2009), have multifactorial origins, depending not only on genetic but also on environmental factors. The most extensively studied neurological disorder regarding epigenetic changes is Rett syndrome. Patients with Rett syndrome exhibit neurodevelopmental defects associated with MeCP2 mutations, which encode methyl CpG binding protein 2 and bind to methylated DNA (Ballestar et al., 2000; Hansen et al., 2010). Other mental retardation disorders are also linked to the disruption of genes involved in epigenetic mechanisms, e.g., alpha thalassemia/mental retardation X-linked syndrome, Rubinstein-Taybi syndrome, and Coffin-Lowry syndrome (Urdinguio et al., 2009). Aberrant DNA methylation followed by abnormal gene expression was first reported as an epigenetic hallmark in cancer (Haffner et al., 2011; Malzkorn et al., 2011). The cytosine base *in situ* to produce 5-methylcytosine (5mC) is catalyzed by DNA methyltransferases (Lian et al., 2012). It is estimated that 5mC accounts for 2%-8% of total cytosines in human genomic DNA and affects a broad range of biological functions, including gene expression, genome integrity maintenance, parental imprinting, X-chromosome inactivation, development regulation, aging, and cancer (Guo et al., 2011; Lian et al., 2012; Szwagierczak et al., 2010). Ten-eleven translocation (TET) proteins, which are the enzymes necessary for demethylating 5mC, display enzymatic activity for the conversion of 5mC to 5-hydroxymethylcytosine (5hmC) (Dahl et al., 2011; Kudo et al., 2012). This oxidized form of 5mC is known as the "sixth base". Its presence adds a layer of complexity to the epigenetic regulation of DNA methylation (Ito et al., 2010; Tahiliani et al., 2009) and it is found in a variety of mammalian cells, particularly in self-renewing and pluripotent stem cells and neuronal cells (Guo et al., 2011; Szwagierczak et al., 2010). Although the biological role of 5hmC is not fully understood at present, it has gained attention due to its cancer biomarker potential (Davis & Vaisvila, 2011). It is assumed that, like 5mC, 5hmC also plays an important role in switching genes on and off.

It is important to illuminate the differences in 5hmC expression in different tissues and cells (Almeida et al., 2012a, b). Of note, 5hmC is particularly abundant in the brain (Globisch et al., 2010; Kriaucionis & Heintz, 2009; Li & Liu, 2011), and accounts for approximately 40% of modified cytosine, suggesting a potential role in neuronal plasticity (Szulwach et al., 2011). Low levels of 5hmC are reportedly associated with brain tumor differentiation and anaplasia (Kraus et al., 2012; Lian et al., 2012). In the mouse cortex and cerebellum, 5hmC is enriched within genes and appears to promote gene transcription (Jin et al., 2011; Song et al., 2011). As an intermediate state of complete DNA demethylation, 5hmC affects functional demethylation by blocking transcriptional repressor binding (Guo et al., 2011; He et al., 2011; Ito et al., 2011; Zhang et al., 2012). Synchronous neuronal activity promotes active DNA demethylation of plasticity-related genes in the mouse brain through TET-mediated formation of 5hmC (Guo et al., 2011). However, it is not clear whether such demethylation solely accounts for the enrichment of 5hmC in the brain. Recent research has reported that DNA methylation dynamics influence brain function and are altered in neurological disorders (Chia et al., 2011). Genome-wide mapping of 5hmC in the mouse hippocampus and cerebellum at different developmental stages indicate an increase with age and gene expression related enrichment in genes implicated in neurodegeneration (Szulwach et al., 2011). In addition, our lab recently revealed that selective changes in 5hmC-mediated epigenetic regulation promote Purkinje cell neurodegeneration in Ataxia-Telangiectasia disease and regulate DNA damage response (Jiang et al., 2015, 2017).

The tree shrew (*Tupaia belangeri chinensis*) belongs to Order Scandentia and is similar to primates in many biological features (Fan et al., 2013; Rockland & Lund, 1982; Xu et al., 2012). Moreover, its high brain-to-body mass ratio makes it a promising non-human primate animal model in brain and biomedical research (Cao et al., 2003; Li et al., 2017). In the present study, we sought to define dynamic changes in 5hmC levels in the tree shrew brain during postnatal brain development and aging, as well as its potential epigenetic regulation. During postnatal development, the levels of 5hmC showed a gradual increase in multiple anatomic structures of the tree shrew brain. In contrast, a significant loss of 5hmC was found in several selective regions of the aged tree shrew brains. Thus, dynamic changes in 5hmC levels suggest its potential epigenetic regulation in the process of brain development and aging.

## MATERIALS AND METHODS

### Animal use and care

Wild-type tree shrews were obtained from the Kunming Primate Research Center, Kunming Institute of Zoology, Chinese Academy of Sciences. All experimental procedures and animal care and handling were performed per the protocols approved by the Institutional Animal Care and Use Committee of the Kunming Institute of Zoology, Chinese Academy of Sciences. For 5hmC immunohistochemistry, P10-, 3-month, 1-, 2-, 4-, and 5-year-old age-matched groups of animals (three males and three females) were used; for dot-blot assay, 3-month, 1-, 2-, and 4-year-old age-matched groups of animals (three males and three females) were used; for *Tet* mRNA quantitative real-time PCR, P10-, 3-month, 1-, 2-, and 4-year-old age-matched groups of animals (three males and three females) were used.

### Tree shrew brain cryostat section preparation

The tree shrews were anaesthetized with ketamine (0.2 mg/g, i.m.) and perfused with phosphate buffer saline (PBS; 137 mmol/L NaCl, 2.7 mmol/L KCl, 10 mmol/L Na_2_HPO_4_, 2 mmol/L KH_2_PO_4_, pH 7.4) after complete anesthesia. After the blood was rinsed off from the body of animal, the perfusion buffer was changed to 4% paraformaldehyde (PFA) fixative solution for 20 min. Immediately, the whole brain was dissected using scissors and fixed with 4% PFA overnight at 4 ℃. The fixed brain tissue was placed in 10, 20, and 30% sucrose solution sequentially for dehydration until it sank, and was then completely immersed with embedding agent optimal cutting temperature compound (OCT, Ted Pella Inc., USA). Finally, 15 μm cryostat brain sections were prepared at -25 ℃ and stored at -80 ℃.

### Immunohistochemistry

For 3, 3'-diaminobenzidine (DAB)/bright field staining, all frozen brain sections were pretreated with 0.3% hydrogen peroxide in methanol for 30 min to inhibit endogenous peroxidase activity, then rinsed in Tris-buffered saline (TBS), and treated with 0.1 mol/L citrate buffer in a microwave at sufficient power to keep the solution at 100 ℃ for 20 min. Sections were cooled in the same buffer at room temperature (RT) for 30 min and rinsed in TBS. Slides were incubated in 10% goat serum in PBS blocking solution for1 h at RT, after which anti-5hmC antibody (Abcam, ab106918, 1:200 dilution) was applied to the sections, followed by incubation at 4 ℃ overnight. The sections were washed three times in TBS before applying the secondary antibody (Vector Laboratories) for 1 h at RT. Afterwards, sections were rinsed three times in TBS. Rinsed sections were then incubated in Vectastain ABC Elite reagent (Vector Laboratories) for 1 h and developed using diaminobenzidine, according to the manufacturer's protocols. The sections were counterstained with hematoxylin, and finally mounted in Permount under a glass cover slip after dehydration. Control sections were subjected to identical staining procedures, except for the omission of the primary antibody.

### Dot-blot analysis

Genomic DNA was isolated from brain tissue using a QIAamp DNA Mini Kit (Qiagene). Dot-blot assays were performed as described previously (Jiang et al., 2015). Briefly, 10 μg of sample DNA was diluted in Tris-EDTA (10 mmol/L, pH 8.0) buffer to 90 μL, with 2 μL of 5 mol/L NaOH buffer then added. After denaturing at 98 ℃ for 10 min, samples were immediately chilled on ice and neutralized with 10 μL of 6 mol/L (NH_4_)_2_Ac to a final DNA concentration at 100 ng/μL. The indicated amount of mixed DNA solution was spotted on a N+-Nylon membrane (Amersham Biosciences) and then crosslinked by UV-x-linker at 0.5 Joule/cm^2^. Blots were incubated in blocking buffer (5% non-fat milk and 1% BSA in PBS-T) for 1 h at RT and then in 5hmC (Active Motif 39769) solution (1:10 000 in blocking buffer) overnight at 4 ℃. Corresponding secondary Ab-HRP (1:5 000 in 5% non-fat milk) was used and blots were detected with the ECL system following the manufacturer's instructions. Methylene blue (0.02% in Na2Ac, pH 5.5) staining was also performed to measure total DNA loading.

### RNA isolation and real-time quantitative PCR (RT-qPCR)

Total RNA was isolated from tree shrew brain tissue using Trizol reagent (Invitrogen) and cDNA was generated with a Fast Quant RT Kit (Vazyme). Real-time qPCR reactions were performed on an ABI PRISM 7700 Sequence Detection System (Applied Biosystems) using SYBR Green reagent (Invitrogen). Expression levels of target genes were analyzed using the comparative cycle threshold (Ct) method, where Ct is the cycle threshold number normalized to that of β-actin. The primers used in this study were: for *Tet1*, Tet1-F 5'-CGGAATCATC CTACACGCCT-3'/Tet1-R 5'-GGACAGTTCATTACCCTCCTTA-3'; for *Tet2*, Tet2-F 5'-ACCCCCTGATTAATAACCCT-3'/Tet2-R 5'-CACCTCCATAAACACCTGAC-3'; and for *Tet3*, Tet3-F 5'-CAAGGCTGAGAACCCACTCA-3'/Tet3-R 5'-CTTTCTCCACTA TTTGTTCGAC-3'. β-actin was applied as the standard for normalization by using β-actin-F 5'-TGCGTGACATCAAGGA GAAG-3'/β-actin-R: 5'-ACCTGACCATCAGGCAACTC-3'.

### Statistics

All data were presented as means±*SE* of a minimum of three replicates. For most analyses, we evaluated statistical differences using the Student's *t*-test. For all analyses, *P* < 0.05 indicated statistical significance.

## RESULTS

### Spatiotemporal-biased increase of 5hmC in tree shrew brains during postnatal development

**Figure 1:**
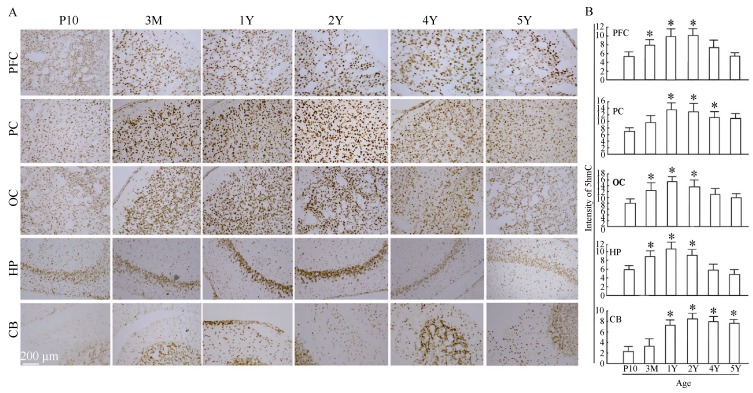
Dynamic changes in 5hmC levels in tree shrew brains during postnatal development and aging

To address if postnatal brain development could be attributed to active DNA demethylation, we examined the 5hmC levels in the prefrontal cortex (PFC), parietal cortex (PC), occipital cortex (OC), hippocampus (HP), and cerebellum (CB) of P10, 3-month, and 1-year-old tree shrew brains using immunohistochemistry. Compared with P10 brains, the intensity of 5hmC in the PFC, PC, OC, and HP neurons showed substantial increases in the 3-month and 1-year-old tree shrew brains, and peaked at 2-years old (Figure 1A, B). However, despite of high abundance of 5hmC in CB neurons in 1-year-old animals, high populations of CB neurons from P10 and 3-month-old animals showed low intensities of 5hmC (Figure 1A, B). The high abundance of 5hmC in adult brains (1-and 2-year-old tree shrews), but not developing brains (P10 and 3-month-old tree shrews), suggests that dynamic changes in 5hmC levels are involved in postnatal brain development. We further analyzed 5hmC-specific immunostaining data and revealed a significant increase in 5hmC intensity from P10 to 1-year, with a 2.31±0.12-fold increase in the PFC (*P* < 0.05), 2.26±0.13-fold increase in the PC (*P* < 0.01), 2.1±0.16-fold increase in the OC (*P* < 0.001), 2.25±0.18-fold increase in the HP (*P* < 0.01), and a 4.5±0.15-fold increase in the CB (*P* < 0.001) (Figure 1B).

### Age-related decrease of 5hmC in tree shrew brains during aging

Although 5hmC-mediated epigenetic regulation plays an important role in brain development, whether it is involved in brain aging remains unclear. To explore if changes in 5hmC levels diminished its dynamics during brain aging, we examined the abundance of 5hmC in the PFC, PC, OC, HP, and CB in 2-, 4-and 5-year-old tree shrew brains. In contrast to the abundance of 5hmC in 2-year-old brains, the intensities of 5hmC were substantially decreased in the PFC, PC, OC, and HP neurons of 4-and 5-year-old tree shrews (Figure 1A-B). In both the cerebral cortices and HP, we found selective loss of 5hmC in most neurons in 4-and 5-year-old tree shrew brains. Nevertheless, decreased 5hmC levels were not significantly observed in CB neurons (Figure 1A-B). The decreased abundance of 5hmC in aged brains (4-and 5-years old), but not adult brains (2-years old), suggests that changes in 5hmC levels are involved in brain aging. We analyzed 5hmC-specific immunostaining and revealed a significant decrease in 5hmC intensities from 2-year-old to 4-and 5-year-old brains, with a 1.75±0.16-fold decrease in the PFC (*P* < 0.05), 1.36±0.14-fold decrease in the PC (*P* < 0.05), 1.6±0.13-fold decrease in the OC (*P* < 0.01), and 1.5±0.17-fold decrease in the HP (*P* < 0.05), but a 0.35±0.08-fold increase in the CB (*P* > 0.05) (Figure 1B).

**Figure 2:**
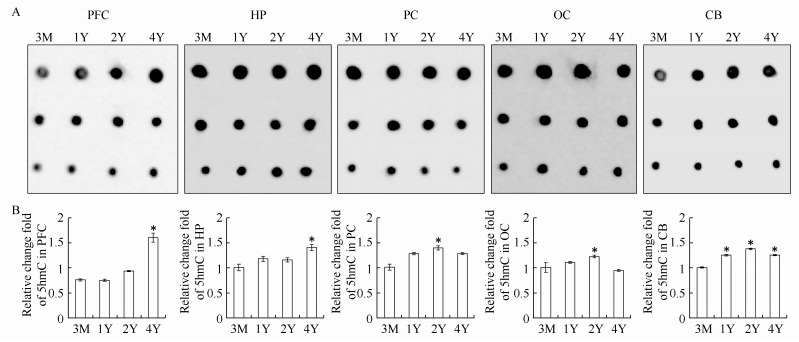
Decreases in 5hmC levels in the prefrontal cortex (PFC) and hippocampus (HP) during aging confirmed by dot-blot assay

To verify dynamic changes in 5hmC levels in tree shrew brains during postnatal brain development and aging, we determined the global changes in 5hmC levels through dot-blot assay. Genomic DNA from the PFC, HP, OC, PC, and CB of 3-month, 1-, 2-, and 4-year-old tree shrew brains was extracted and sonicated for examination. As expected, the levels of 5hmC increased from the P10 to the 2-year-old brains in five regions during postnatal brain development (Figure 2A, B). In contrast, the levels of 5hmC in the aged brains showed a slight decrease in the PC, OC, and CB, but not in the PFC or HP (Figure 2A, B). This indicates that selective reduction in 5hmC levels might play a distinct role in different brain compartments during aging.

### Differential patterns of *Tet* mRNA expression in tree shrew brains during development and aging

**Figure 3:**
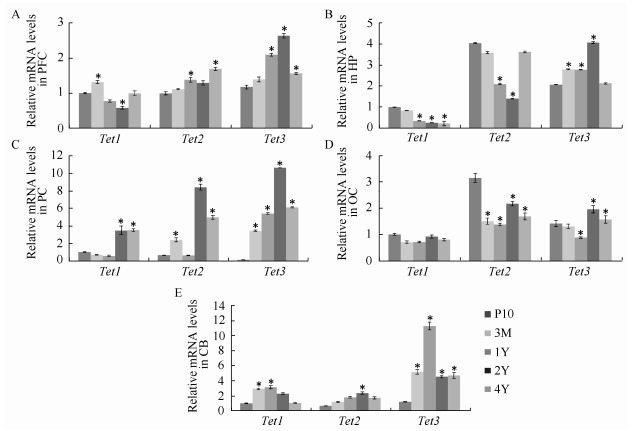
Expression patterns of *Tet* genes in tree shrew brains during postnatal development and aging

The TET family enzymes, including TET1, TET2, and TET3, are responsible for oxidizing 5mC into 5hmC. To test if changes in 5hmC levels were directly associated with levels of *Tet* mRNA expression in tree shrew brains during postnatal development and aging, we examined their expression patterns. Total RNA from the PFC, HP, OC, PC, and CB of P10-, 3-month, 1-, 2-, and 4-year-old tree shrew brains were extracted, followed by RT-qPCR. In the PFC, *Tet3* mRNA expression increased from P10-to 2-years old (2.15±0.11-fold, *P* < 0.05), and then decreased at 4-years old. This is consistent with the patterns of changes in 5hmC levels described above. However, *Tet1* and *Tet2* mRNA expressions exhibited little change during postnatal development in the PFC (Figure 3A). The mRNA levels of *Tet3* showed an exclusive increase in the HP (~2-fold from P10-to 2-years old) (Figure 3B). In contrast, mRNA levels of *Tet1* and *Tet2* in the HP significantly decreased during postnatal brain development (from P10-to 2-years old) (Figure 3B). Interestingly, the mRNA levels of all three *Tet genes* in the PC, and *Tet2/Tet3* in the OC showed significant increases during postnatal development (Figure 3C, D). Lastly, while mRNA levels of *Tet1* and *Tet2* were upregulated in the CB during postnatal development and aging, the level of *Tet3* mRNA remained quite stable (Figure 3E).

## DISCUSSION

DNA methylation/demethylation-mediated epigenetic regulation is involved in the processes of brain development and aging (Hutnick et al., 2009; Wilson et al., 1987). Abnormal alterations in this regulatory system leads to changes in brain function (Jiang et al., 2015; Szulwach et al., 2011); however, the underlying mechanisms remain uncertain in many biological processes and disease states. Loss of DNA methylation can occur through passive or active demethylation (Tahiliani et al., 2009). In active processes, the 5mC methyl group is removed by an enzymatic process. It is proposed that this mechanism requires the action of TET dioxygenases, which hydroxylate 5mC to 5hmC so that it can be deaminated and subsequently repaired by the base excision repair system (Chaudhry & Omaruddin, 2012; Kriaucionis & Heintz, 2009). As a novel epigenetic hallmark, the levels of 5hmC in genomic DNA vary significantly depending on cell type (Guo et al., 2011; Kriaucionis & Heintz, 2009).

In the present study, we observed and assessed dynamic changes in 5hmC levels in the tree shrew brain during postnatal development and aging. We found that the levels of 5hmC in the tree shrew brains increased in multiple anatomic structures during postnatal development, whereas a significant decrease in 5hmC abundance in aged brain neurons was found in the cerebral cortices and hippocampus, but not in the cerebellum. Previous research has reported that 5hmC levels increase in the neurons of the mouse hippocampus and cerebellum during postnatal brain development (Szulwach et al., 2011), similar to the change patterns of the tree shrew brain observed in this study. These findings suggest that the dynamic change in 5hmC levels is an epigenetic hallmark and potential key factor in regulating the processes of brain development and aging. In addition, dynamic changes in 5hmC levels in different neural cell types are different, and its mediated epigenetic regulation during brain development could be different to its role in brain aging.

Brain function is linked to two significant factors: (1) processing of normal, usually abundant, proteins, e.g., neurotransmitter receptors and ion channels; and (2) maturation and aging. Age is not disease-specific, but is a prerequisite in brain development and aging. Our data indicate that understanding abnormal alteration in 5hmC during brain aging will be a useful epigenetic basis to explore the consequences of aging. We revealed that the dynamic pattern of changes in 5hmC levels in tree shrew brains predicts a possible link between DNA demethylation and brain development and aging. This novel perspective significantly broadens the search for new explanations of the complex biology of brain development and aging in tree shrews. The present study will help deepen our understanding of whether abnormal changes in 5hmC levels are epigenetic hallmarks of age-related neurodevelopmental and neurodegenerative disorders, and how changes in 5hmC levels in specific types of neurons produce a broad array of physiological and neurological symptoms.

We presented original data and research on the tree shrew brain, but recognize that our approach entails significant challenges. In the future, we will expand our observations in deciphering the mechanisms of the dynamic changes in 5hmC levels and provide new information about how the 5hmC-mediated regulatory system functions in brain development and aging.

## ACKNOWLEDGEMENTS

We are grateful to De-Wei Jiang for critical reading of the manuscript.
